# The triglyceride-glucose index and risk of cognitive impairment: a systematic review and meta-analysis with inclusion of two national databases

**DOI:** 10.3389/fneur.2024.1496871

**Published:** 2024-11-29

**Authors:** Ying Yang, Pai Peng, Huadong Huang, Yanan Zhao, Yating Li, Xiao Xu, Shixie Jiang, Yanrong Yang, Gaofeng Pan, Yanting Wen, Dan Wu, Shanping Chen, Lei Feng, Tangming Peng, Jiang Wang, Zheng Li

**Affiliations:** ^1^Department of Neurology, Geriatric Diseases Institute of Chengdu/Cancer Prevention and Treatment Institute of Chengdu, Chengdu Fifth People’s Hospital (The Second Clinical Medical College, Affiliated Fifth People’s Hospital of Chengdu University of Traditional Chinese Medicine), Chengdu, China; ^2^School of Computer Science and Technology, Chongqing University of Postsand Telecommunications, Chongqing, China; ^3^School of Clinical Medicine, Jinggangshan University, Ji'an, Jiangxi Province, China; ^4^Online Collaborative Research Center for Evidence-Based Medicine Ministry of Education, Jinggangshan University Branch, Ji'an, Jiangxi Province, China; ^5^School of Basic Medicine, Jinggangshan University, Ji'an, Jiangxi Province, China; ^6^State Key Laboratory of Cardiovascular Diseases, Shanghai East Hospital, School of Medicine, Tongji University, Shanghai, China; ^7^Department of Psychiatry, University of Florida College of Medicine, Gainesville, FL, United States; ^8^Department of Ultrasound Medicine, Chengdu Fifth People’s Hospital, Chengdu, Sichuan Province, China; ^9^Department of Geriatrics, Chengdu Fifth People’s Hospital, Chengdu, Sichuan Province, China

**Keywords:** the triglyceride and glucose (TyG) index, cognitive impairment(CI), prevalence, elderly people, inpatients, Vascular Cognitive Impairment (VCI)

## Abstract

**Background:**

To investigate the relationship between the triglyceride and glucose (TyG) index and cognitive impairment (CI).

**Methods:**

Five authoritative databases were systematically searched for potentially relevant studies on ‘TyG index’ and ‘CI’ from inception to 27 April 2024. Two representative databases from the United Kingdom and United States were also included. We used the PICOS criteria to select available articles. All data was combined to compute Odd Ratios (ORs).

**Results:**

15 studies were included in the meta-analysis (participants: 5604303). The pooled effect sizes demonstrate that individuals with a high TyG index exhibit a significantly elevated risk of CI compared to those with a low TyG index (OR = 2.16, 95%CI: 1.51; 3.08, *p* < 0.001). The subgroup analysis showed that inpatients with a high TyG index exhibited an increased risk of CI (OR = 4.56, 95%CI: 3.09; 6.74, *p* < 0.001). Furthermore, the risk of developing distinct types of CI differed significantly [CI: OR = 1.64, 95% CI: 1.29; 2.07, *p* < 0.001; Vascular Cognitive Impairment (VCI): OR = 5.39, 95% CI: 3.33; 8.70, *p* < 0.001].

**Conclusion:**

A positive correlation exists between the TyG index and risk of CI, which has potential value in optimizing CI risk stratification among elderly people, especially those hospitalized.

**Systematic review registration:**

https://www.crd.york.ac.uk/prospero/, identifier CRD42023450336.

## 1 Introduction

Cognitive impairment (CI) is an aging-related syndrome and evaluated through the exacerbation of memory, thinking, language, and executive function in clinical settings (1). The spectrum of CI in the elderly can be classified as normal cognitive decline with aging to subjective cognitive decline (SCD) to mild cognitive impairment (MCI) to dementia (2–5). Statistical data indicate that in the early 21st century, over 35 million people have suffered from Alzheimer’s disease (AD), which is one of the most common causes of dementia and is a major socioeconomic burden worldwide (6, 7). Reversing brain tissue degradation and cognitive decline is challenging, especially for older adults with lower education, lower MMSE scores, the APOEe4 allele, or chronic diseases. Early intervention is essential once CI is detected to prevent further decline or irreversible dementia. Thus, the emphasis on early prevention and management of CI is more critical than treatment itself (8–10).

Early identification of CI is of paramount importance, despite the challenges it presents (11–13). CI is one of the most critical symptoms to watch for in the early stages of AD, but it is not exclusive to AD (13–15). It can also be associated with a variety of other conditions, such as other types of neurodegenerative diseases (16, 17), vascular dementia (18), and endocrine disorders (19, 20). The irreversible damage to the nervous system caused by these diseases underscores the pivotal role of early diagnosis in slowing the progression of the disease. Currently, we employ a range of tools to assist in the early detection of CI, including neuropsychological assessments, genetic screening, brain imaging techniques, and biomarkers in serum or cerebrospinal fluid (4, 21, 22). While these methods face certain clinical debates due to issues such as insufficient accuracy (4), low specificity (21, 23, 24), invasiveness (25), and the involvement of ionizing radiation (22), and the difficulty in precisely achieving early diagnosis of non-AD from different types and pathological mechanisms (22), these methods continue to be valuable instruments for detecting CI (26). Particularly, blood-related markers stand out for their ease of access, minimal risk, and the elimination of human subjectivity in the testing process, making them worthy of further in-depth research.

The Triglyceride Glucose (TyG) index, calculated as ln [(fasting triglycerides (mg/dL) × fasting glucose (mg/dL))/2], serves as a simple, cost-effective, and easily replicable marker for insulin resistance (IR) (27). IR is associated with neurotoxicity and negatively impacts cognitive functions, particularly learning and memory (28, 29). The TyG index is sensitive in identifying metabolic syndrome and is linked to CI through potential mechanisms such as endothelial dysfunction, blood–brain barrier (BBB) dysfunction, oxidative stress, and inflammation due to insulin response failure in brain cells (30, 31). Studies suggested that IR may also disrupt brain metabolism and neurodegenerative processes leading to CI by affecting the release of neurotransmitters and receptor activity, thereby impacting hippocampal synaptic plasticity (1, 32, 33).

Based on these seminal studies, there are strong implications and suggestions that the TyG index may be independently associated with CI. To investigate this, the objective of our study was to synthesize existing prospective evidence on the relationship between TyG index and CI, supplemented by original individual-level data obtained from nationally representative samples from England and the United States.

## 2 Methods

The study protocol was registered on PROSPERO (International Prospective Register of Systematic Reviews) with registration number CRD42023450336. The URL is https://www.crd.york.ac.uk/PROSPERO/#searchadvanced. The systematic review and meta-analysis was conducted according to the Preferred Reporting Items for Systematic Reviews and Meta-Analyses (PRISMA) guidelines (34).

### 2.1 Literature search

We searched PubMed, Embase, Medline, Web of Science, and the Cochrane Library from inception to 12 January 2023 for potentially relevant studies by using the search terms: (1) Exposure: For the TyG index: Triglyceride glucose index or TyG index or TyG. (2) Outcomes: For CI: Cognitive Dysfunction or Cognitive Dysfunctions or Dysfunction*, Cognitive or Cognitive Impairment* or Impairment*, Cognitive or Cognitive Disorder*, Disorder*, Cognitive, or Cognitive Decline* or Mental Deterioration* or Dementia or Dementia* or Amentia*. Furthermore, we searched glucose and triacylglycerol separately to capture as many studies as possible that met the inclusion criteria.

### 2.2 Data updating

We searched the five databases as mentioned before from 12 January 2023 to 27 April 2024 to update our data.

### 2.3 Study selection

The selection was performed with Endnote X9. After removing duplicates, we searched several keywords to exclude undesired study types including reviews, conference abstracts, and commentaries. The initial screening was performed by checking titles and abstracts, with full-text screening performed. All the above steps were completed by two reviewers independently (YY and PP), and any discrepancies were resolved by a third reviewer (PH).

We used the PICOS method to establish our inclusion criteria, which is as follows: (1) Participants: Adults (age > 18 years) in the whole population (without CI) or adult patients with CI. (2) Exposure and comparator: high versus low TyG index level. (3) Outcomes: the occurrence or prediction of CI in the whole population and the occurrence or prediction of other clinical outcomes in patients with CI (other clinical outcomes include: death, complications, co-morbid disease, etc.) (4) Type of studies: cohort study, case–control study, and cross-sectional study.

Our exclusion criteria included: (1) Studies that were not written in English, (2) Conference abstracts, case reports, commentaries, letters to the editor, reviews, and preclinical studies. (3) Studies with insufficient data, such as lack of clear CI diagnostic criteria or inability to calculate TyG index.

### 2.4 Data extraction and quality assessment

The data of articles were extracted independently by two authors (YY and HDH) using standardized data abstraction sheets. Extracted data included the first author, year of publication, study design, country, male proportion, mean age, sample size, data source, TyG index condition, assessment of CI, and type of cognitive disorder. Furthermore, we used the data from the National Health and Nutrition Examination Survey (NHANES) and the English Longitudinal Study of Aging (ELSA) to enlarge our study results, and the details of these data have been described in Supplementary file 1.

We used the Newcastle–Ottawa Scale (NOS) to assess the quality of cohort studies and case–control studies. This scale ranges from 1 to 9 points to assess the selection of articles, comparability of the groups, and outcomes. When the total score is >6, it is considered to be of high quality (35). The Agency for Healthcare Research Quality (AHRQ) was adopted to evaluate the included cross-sectional studies. The AHRQ consists of 11 items, where each item can be answered with a “yes,” “no,” or “not reported.” A score of 1 is given for a “yes” answer, while 0 is given for a “no” or “not reported” answer. A study is considered high quality if it scores 8–11. Two authors independently completed the assessment of quality (YY and XX).

### 2.5 ELSA and NHANES databases

We used the data from ELSA and NHANES to supplement the studies identified through the literature database searches.

The ELSA is an ongoing representative cohort of a national sample of men and women aged 50 years and older living in England (36). Comparisons of the socio-demographic characteristics of participants against results from the national census indicate that the sample is broadly representative of the English population (37). It began in 2002 and includes follow-up data every 2 years to measure the changes. So far, it has conducted 10 waves; we used the data from waves 4 and 6 which have the data we need (the data from waves 1,3,5 and 7 lacked blood test results, so the TyG index cannot be calculated. From wave 8 onwards, a health visit was carried out on half the sample every 2 years. Considering the avoidance of severe data duplication, only the previous data from the database was used). Participants gave informed consent to participate in the study, and ethical approval for all the ELSA waves was granted from NHS Research Ethics Committees under the National Research and Ethics Service (NRES).

The NHANES is the most in-depth survey designed to evaluate the health and nutritional status of adults and children in the United States of America (38). This program began in the early 1960s, and has become a continuous program focusing on a variety of health and nutrition measurements to meet emerging needs in 1999. The survey examines a sample of approximately 5,000 persons with national representation each year. We used the data from 2011 to 2014.

### 2.6 Statistical analysis

Stata version 16.0 and R statistical language version R 4.3.1 were independently used to analyze the extracted data by two authors (YY and PP). In analyzing data from ELSA and NHANES, we used a multivariable logistic regression to compute Odds Ratios (ORs) with accompanying 95% Confidence Intervals (CIs) to assess the association of the TyG index with CI with three different models. Model 1 was not adjusted, Model 2 was adjusted for age and gender, and Model 3 was adjusted for age, gender, chronic kidney disease (CKD), smoking, alcohol, and dyslipidemia. For selected articles, we used a random effect model to calculate pooled ORs and their corresponding 95% CIs to estimate the association between the TyG index and CI. An I^2^ statistic was used to quantify the heterogeneity among studies. While 25% represented low heterogeneity, 50 and 75% expressed moderate and high heterogeneity, respectively (39). The TyG index was calculated as follows: TyG = Ln (TG [mg/dL] × fasting glucose [mg/dL]/2). According to the data type of TyG index and the evaluation methods of CI given in the included studies, we divided all the studies into four groups and pooled the effect size separately. Furthermore, the cross-sectional data collected from the two national databases were combined with the data from literature search-included cross-sectional studies.

Subgroup analyses were carried out to identify the difference between subgroups and potential sources of the observed heterogeneity. *p* < 0.05 was regarded as statistically significant. We used sensitivity analyses to evaluate the robustness of results by omitting studies one after another. The publication bias of this review was assessed with the combination of funnel plots and Begg’s test (40).

## 3 Results

### 3.1 Selection details

**Figure 1:**
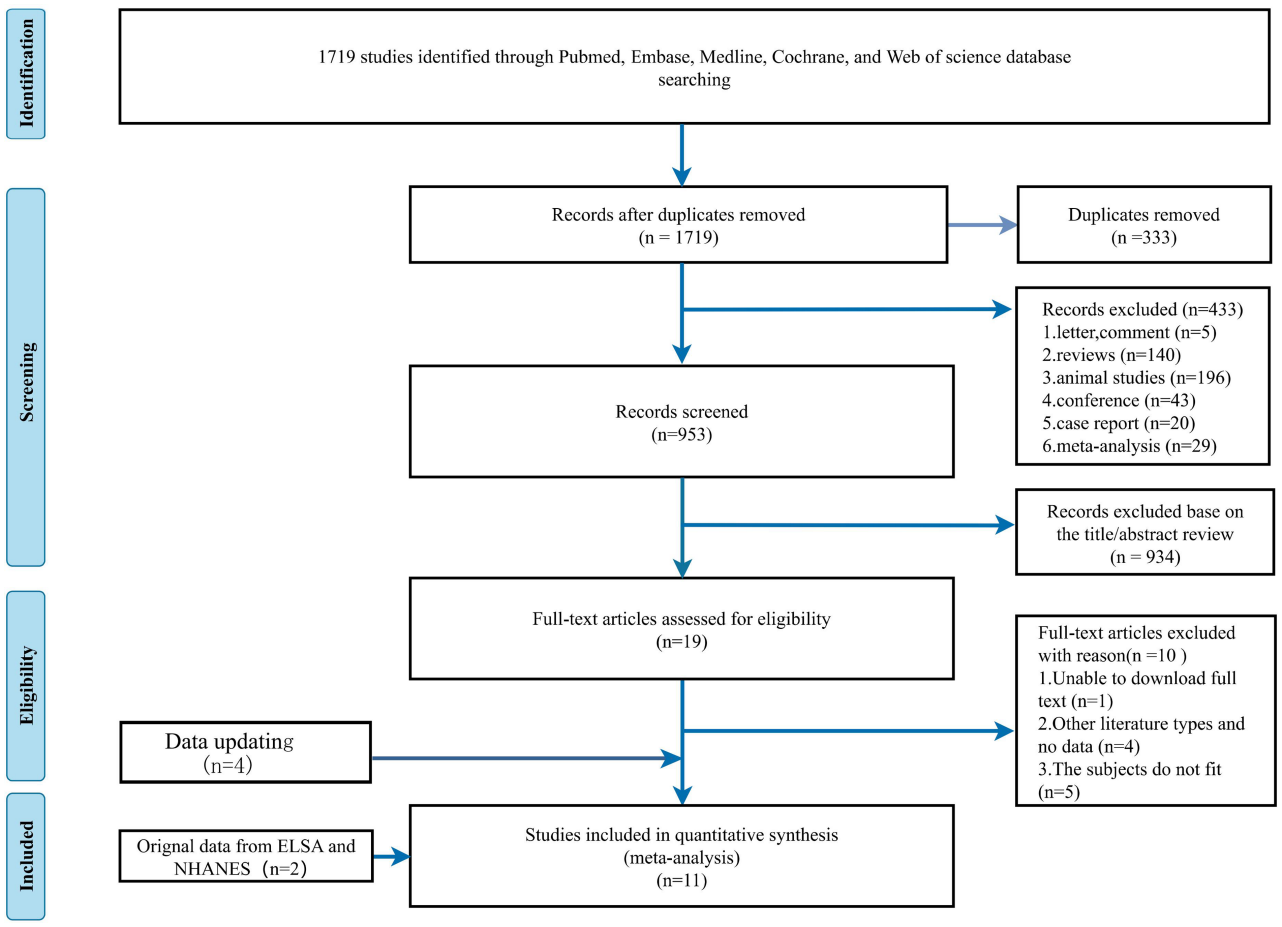
The flow diagram of studies selection.

Our search strategy initially identified 1719 potential articles. 333 articles were removed as duplicated files, and we excluded 433 non-related articles: letters to the editor (*n* = 4), commentary (*n* = 1), reviews (*n* = 140), animal studies (*n* = 196), conference abstracts (*n* = 43), case reports (*n* = 20), and meta-analysis (*n* = 29). After screening based on title and abstract, we further narrowed 934 articles to 19 articles. We incorporated two additional datasets, the ELSA and NHANES. By reviewing the full text of the preliminary screening literature, we included 11 papers that met our inclusion criteria and excluded 10 other studies. One study was excluded as the full text was inaccessible, 4 studies were other types of literature or included no data, and 5 were unsuitable studies because the subject did not fit due to lacking appropriate subject types. An update with four new articles was made on 27 April 2024. A total of 15 studies were ultimately included. The study selection process is presented in Figure 1.

### 3.2 Included studies

The essential characteristics of all included observational articles are summarized in Table 1. After screening, a total of 15 papers were included in our meta-analysis, comprising 7 cohort studies (1, 28, 30, 41–44), 6 cross-sectional studies (29, 33, 36, 38, 45, 46), and 2 case–control studies (31, 32). In accordance with Wang’s 2022 original study, we conducted a gender-based data analysis to investigate potential disparities in the relationship between the TyG index and CI (41). These participants originated from various countries, including South Korea (28), China (1, 29–31, 33, 41, 42, 46), Saudi Arabia (43), the United States of America (38, 44, 45), England (36), Mexico (32), and data from two national databases, NHANES and ELSA. Of these literature, nine used TyG index quartiles (1, 28, 31, 33, 36, 38, 41, 44, 45), four followed a TyG index tertiles (29, 30, 42, 43), two applied TyG index dichotomized and the remaining studies did not mention specific TyG index conditions (32, 46). A total of 5,604,303 participants were included in these studies, with a mean age between 40.8 and 90.7 years old, comprising both community populations and hospital inpatients. The follow-up period for the cohort studies ranged from 4 to 13.8 years, and sample sizes ranged from 135 to 5,586,048. Among the included studies, four adopted the Mini-Mental State Examination (MMSE) to evaluate CI among participants (1, 30, 32, 33), two used the Montreal Cognitive Assessment (MoCA) (31, 42); The remaining studies used different cognitive assessment methods, including WRT plus MST (Word Recall Test and Mental Status Test) (41), the International Classification of Diseases-10 (ICD-10) criteria (28), National Institute on Aging-Alzheimer’s Association Workgroups (NIAAA) criteria (29), clinical criteria for AD diagnosis, Diagnostic and Statistical Manual of Mental Disorders, Fourth Edition, criteria (DSM-IV) (46), Consortium to Establish a Registry for Alzheimer’s disease (CERAD) (45), National Institute of Neurological and Communicative Disorders and Stroke and the AD and Related Disorders Association (NINCDS-ADRDA) (44), Digital Symbol Substitution Test (DSST) (38), and Cognitive comprehensive assessment (i.e., immediate word recall test, delayed word recall test, and orientation to date test) (36).

**Table 1:** Characteristics of included studies.

Study	Country	Study design	Male%	Age(mean ± SD);years	Sample size	Study duration	Participants	TyG index condition	Cognitive impairment assessment method	Type of cognitive disorder
Hong 2021	Korea	Retrospective cohort study	50.69%	TyG Q1: 40.77 ± 13.3 TyG Q2: 44.08 ± 13.24 TyG Q3: 46.48 ± 13.12 TyG Q4: 48.49 ± 12.95	5,586,048	7.21 years	National Health Screening program in 2009 from NHID	TyG I	ICD-10	Dementia, Alzheimer’s disease
Tong 2022	China	Cross-sectional study	54.40%	Mean age: 58	517	—	Type 2 diabetic patients hospitalized in the Department of Endocrinology, the First Affiliated Hospital of Harbin Medical University	TyG III	NIAAA	Mild cognitive impairment
Li 2022	China	Prospective cohort study	40.88%	53.48 ± 8.47	1774	4 years	The Jidong Cognitive Impairment Cohort Study (CICS)	TyG I	MMSE	Cognitive decline
Teng 2022	China	Retrospective cohort study	79.61%	70.6 ± 6.1	308	4.5 years	A total of 308 eligible patients with Type 2 Diabetes Mellitus who were admitted to Hebei General Hospital	TyG III or TyG IV	MMSE	Cognitive impairment
Wang 2022	China	Prospective cohort study	46.7%	59.84 ± 8.72	2062	4 years	Data from the China Health and Retirement Longitudinal Study	TyG I	WRT plus MST	Cognitive decline
Jiang 2021	China	Case–control study	43.01%	VCI: 68.93 ± 10.88 Non-VCI: 65.61 ± 12.97	280	—	Patients with CSVD who were hospitalized at the Second Affiliated Hospital of Nantong University	TyG I	MOCA	Vascular cognitive impairment
Faqih 2021	Saudi Arabia	Retrospective cohort study	52.76%	80.5 ± 10.2	354	9 years	Patients from King Abdulaziz Medical City, Jeddah, Ministry of National Guards-Health Affairs	TyG III	AD was defined according to clinical criteria for AD diagnosis	Alzheimer’s disease
Weyman-Vela 2022	Mexico	Case–control study	18.5%	72.8 ± 6.2	135	—	Participants in the study were from Durango	TyG II	MMSE	Mild cognitive impairment
Ma 2023	China	Cross-sectional study	40.3%	58.07 ± 9.25	1,484	—	Huyi District, Xian, Shaanxi Province, China	TyG I or TyG IV	MMSE	Cognitive impairment
Tian 2023	China	Cross-sectional study	42.87%	71.76 ± 5.52	5,199	—	Multimodal Interventions to Delay Dementia and Disability in rural China	TyG II	DSM-IV	All-cause dementia
Cheng 2024	China	Prospective cohort study	61.7%	67.30 ± 13.41	313	1 year 5 months	Patients from inpatient department of the First People’s Hospital of Yancheng, China.	TyG III	MOCA	Post-stroke cognitive impairment (PSCI)
Sun 2023	America	Prospective cohort study	46.7%	63.0 ± 8.20	2,170	13.8 years	Participants from the Framingham Heart Study (FHS) Offspring cohort	TyG I or TyG IV	NINCDS-ADRDA	Dementia, Alzheimer’s disease
Wei 2023	America	Cross-sectional study	47%	68.62 ± 6.49	661	—	Participants in the National Health and Nutrition Examination Survey (2011–2012, 2013–2014)	TyG I or TyG IV	CERAD	low cognition function
NHANES	USA	Database	49.17%	69.67 ± 6.78	1,391	—	Participants in the National Health and Nutrition Examination Survey(2011–2014)	TyG I	DSST	Cognitive decline
ELSA	England	Database	42.50%	62.88 ± 6.36	1,607	—	Participants in Wave 2, Wave 4 and Wave 6 of the English Longitudinal Study of Aging	TyG I	Immediate word recall test, delayed word recall test and orientation to date test	Cognitive impairment

TyG index, Triglyceride glucose index; NIAAA, National Institute on Aging-Alzheimer’s Association Workgroups; MOCA, Montreal cognitive Assessment; MMSE, Mini Mental State Examination; ICD-10, International Classification of Diseases-10; WRT, word recall test; MST, mental status test; DSST, digital symbol substitution test; DSM-IV, Diagnostic and Statistical Manual of Mental Disorders, Fourth Edition, criteria; CERAD, Consortium to Establish a Registry for Alzheimer’s disease; NINCDS-ADRDA, National Institute of Neurological and Communicative Disorders and Stroke and the AD and Related Disorders Association; TyG I, TyG index quartiles; TyG II, TyG index dichotomized; TyG III, Tyg index tertiles; TyG IV, TyG index (Uncategorized, count data); AD, Alzheimer’s disease. NHANES, National Health and Nutrition Examination Survey; ELSA, The English Longitudinal Study of Aging; CSVD, cerebral small vessel disease.

### 3.3 Quality assessment

The quality assessments of cohort and case–control studies used the NOS. Studies with a NOS score of ≥7 (1, 28, 30, 32, 41–44) were considered high-quality. Eight studies obtained a score of ≥7, and one was assessed with a score of 6 (31). The Agency for AHRQ scale was employed for the assessment of cross-sectional studies (29, 33, 36, 38, 45, 46). We regarded studies with AHRQ scores ≥8 as high quality according to the inclusion of 11 criteria items. Among the four cross-sectional studies included, two obtained a score of 6 (29, 45), one received a score of 7 (46), and the remaining study achieved a score of 8 (33). Two researchers (YY and XX) independently evaluated different types of studies using appropriate scales.

### 3.4 The impact of the TyG index on CI

#### 3.4.1 Meta-analysis of studies

**Figure 2:**
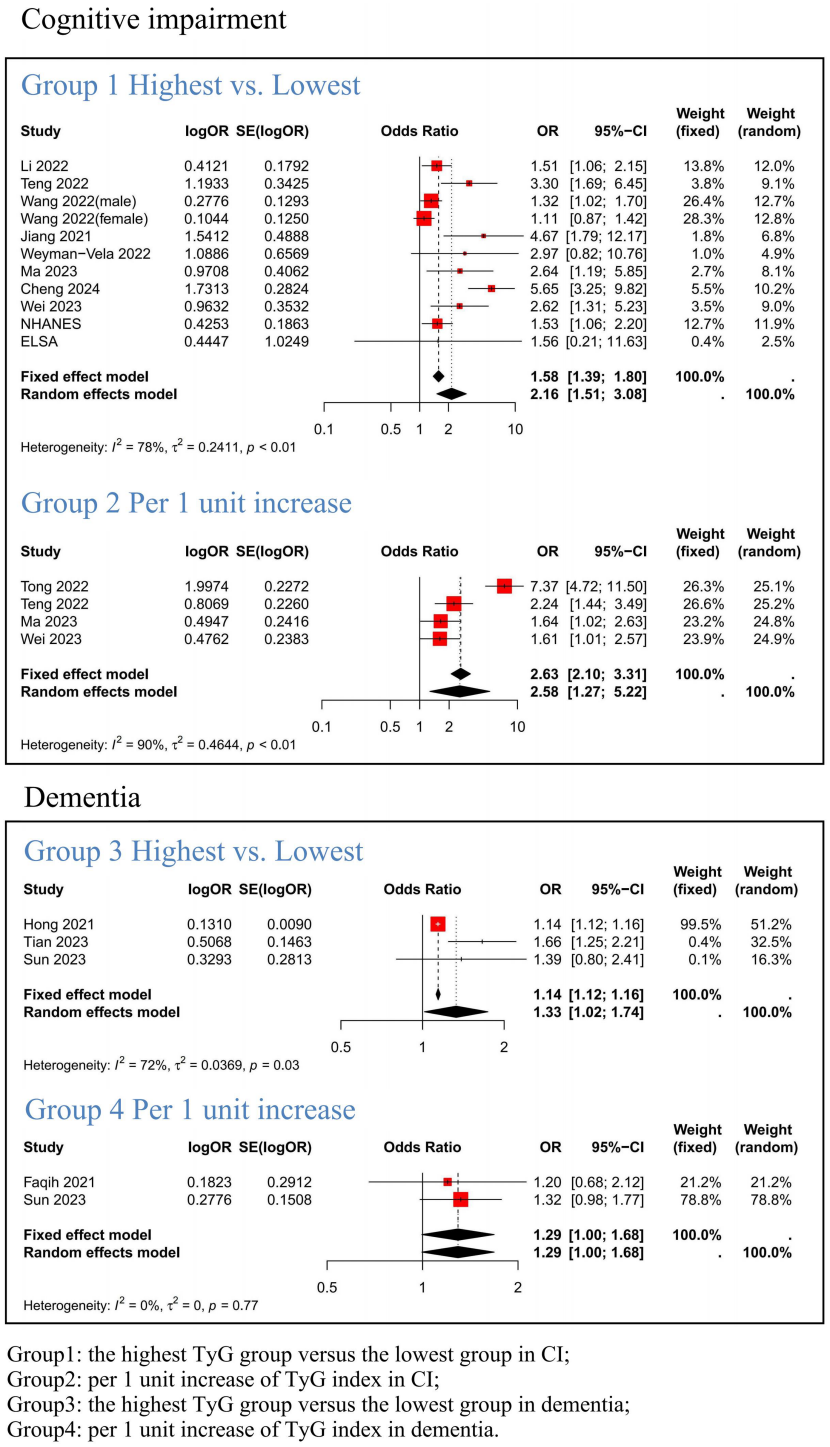
Meta-analysis results link the TyG index to Cognitive Impairment and Dementia. Group1: the highest TyG group versus the lowest group in CI; Group2: per 1 unit increase of TyG index in CI; Group3: the highest TyG group versus the lowest group in dementia; Group4: per 1 unit increase of TyG index in dementia.

We conducted a meta-analysis of 15 studies to investigate the association between TyG index and CI (encompassing NHANES and ELSA databases). Given the substantial heterogeneity observed across the included studies, we employed a random-effects model to estimate the pooled ORs. The synthesized findings are presented in Figure 2. Effect sizes of the four groups were analyzed separately to further investigate the association between TyG index and CI as well as dementia. Group 1 demonstrated that a high TyG index was significantly correlated with CI (OR = 2.16, 95% CI: 1.51; 3.08, *p* = <0.001). In Group 2, it was found that the risk of CI increased by 158% per 1 unit increase in TyG index when treated as a continuous variable (OR = 2.58, 95% CI: 1.27; 5.22, *p* = 0.009). Group 3 indicated an association between elevated TyG index levels and dementia (OR = 1.33, 95% CI: 1.02; 1.74), while Group 4 revealed a 29% increase in dementia risk per 1 unit increase in TyG index when considered as a continuous variable (OR = 1.29, 95% CI: 1.00; 1.68, *p* = 0.001).

#### 3.4.2 Subgroup analysis

In light of the higher heterogeneity observed, we conducted subgroup analyses on studies of CI that were not dementia, including 11 (Table 2) and 4 articles (Supplementary file 4) for subgroup analysis based on different methods of evaluating the TyG index, to further clarify the association between the TyG index and CI.

**Table 2:** The results of subgroup analysis of the correlation between TyG index and cognitive impairment.

Variable	N	Meta-analysis results	P(z-text)	Heterogeneity
Total	11*	2.16 (1.51, 3.08)	<0.001	I^2^ = 78.0%, *p* < 0.01
Mean age				
≥65 years	6	3.07 (1.84, 5.13)	<0.001	I^2^ = 71.6%, *p* = 0.003
<65 years	5	1.33 (1.09, 1.62)	0.004	I^2^ = 25.7%, *p* = 0.250
Study population				
Community-dwelling participants	8	1.47 (1.21, 1.80)	<0.001	I^2^ = 36.0%, *p* = 0.142
Hospital participants	3	4.56 (3.09, 6.74)	<0.001	I^2^ = 0.0%, *p* = 0.479
Region				
Asia	7	2.22 (1.44, 3.40)	<0.001	I^2^ = 85.5%, *p* = 0.000
North America	3	1.87 (1.26, 2.78)	0.002	I^2^ = 18.9%, *p* = 0.292
Europe	1	1.56 (0.21, 11.63)	0.664	–
Cognitive impairment assessment				
MoCA	2	5.39 (3.33, 8.70)	<0.001	I^2^ = 0.0%, *p* = 0.736
MMSE	4	2.22 (1.41, 3.50)	0.005	I^2^ = 43.9%, *p* = 0.148
Other	5	1.37 (1.10, 1.71)	0.001	I^2^ = 36.9%, *p* = 0.175
Study design				
Longitudinal study	7	2.22 (1.43, 3.45)	<0.001	I^2^ = 85.2%, *p* = 0.000
Prospective cohort study	4	1.77 (1.08, 2.90)	0.023	I^2^ = 89.4%, *p* = 0.000
Retrospective cohort study	1	3.30 (1.69, 6.45)	<0.001	–
Case–control study	2	3.97 (1.84, 8.57)	<0.001	I^2^ = 0.0%, *p* = 0.580
Cross-sectional study	4	1.82 (1.35, 2.45)	<0.001	I^2^ = 0.0%, *p* = 0.425
Type of cognitive disorder				
Cognitive impairment	9	1.64 (1.29,2.07)	<0.001	I^2^ = 53.3%, *p* = 0.029
Vascular cognitive impairment	2	5.39 (3.33,8.70)	<0.001	I^2^ = 0.0%, *p* = 0.736

OR, Odds Raito; TyG index, Triglyceride glucose index; MOCA, Montreal cognitive Assessment; MMSE, the Mini Mental State Examination; ICD-10, International Classification of Diseases-10. *As described in the manuscript, two databases were seen as two cross-sectional studies, therefore 10 studies described the association between TyG index and cognitive impairment, but one study listed effect sizes for TyG index and cognitive impairment in men and women separately, so a total of 11 data were ultimately included in the meta-analysis.

Subgroup analysis based on age revealed that participants ≥65 years old with a higher TyG index had a significantly increased risk of developing CI (OR = 3.07, 95% CI: 1.84; 5.13, *p* < 0.001) (30–32, 38, 42, 45). Additionally, those <65 years old with an elevated TyG index also showed a significant increase in CI risk, although the effect size was moderate (OR = 1.33, 95% CI: 1.09; 1.62, *p* = 0.004) (1, 33, 36, 41).

The pooled effects across different clinical settings revealed that hospitalized participants with a high TyG index had a significantly increased risk of CI (OR = 4.56, 95% CI: 3.09; 6.74, *p* < 0.001) (30, 31, 42). In contrast, the relationship was less pronounced among community-dwelling participants (OR = 1.47, 95% CI: 1.21; 1.80, *p* < 0.001) (1, 32, 33, 36, 38, 41, 45).

Regional subgroup analyses revealed significant regional variations in the risk of CI among participants with a high TyG index [Asia: OR = 2.22, 95% CI: 1.44; 3.40, *p* < 0.001; (1, 30, 31, 33, 41, 42); North America: OR = 1.87, 95% CI: 1.26; 2.78, *p* = 0.002; (38, 45); Europe: OR = 1.56, 95% CI: 0.21; 11.63, *p* = 0.664; (36)].

In addition, we also conducted subgroup analyses for the assessment of CI, type of cognitive disorder, and study design, respectively. The results revealed variations in the definition of CI across different rating scales. Furthermore, the risk of developing distinct types of CI differed significantly [CI: OR = 1.64, 95% CI: 1.29; 2.07, *p* < 0.001; (1, 30, 32, 33, 36, 38, 41, 45); Vascular Cognitive Impairment (VCI): OR = 5.39, 95% CI: 3.33; 8.70, *p* < 0.001; (31, 42)].

Regarding analysis of subgroups by research design, they were classified as longitudinal or cross-sectional studies with corresponding effect values [longitudinal study: OR = 2.22, 95% CI: 1.43; 3.45, *p* < 0.001; (1, 30–32, 41, 42); cross-sectional study: OR = 1.82, 95% CI: 1.35; 2.45, *p* < 0.001; (33, 36, 38, 45)]. The subgroup analyses are presented in Table 2.

Group 2 included the TyG index as a continuous variable and examined the associations of age and study population with CI. In terms of study population subgroups, the results were consistent with those observed in Group 1[Community-dwelling participants: OR = 1.62, 95% CI: 1.17; 2.27, *p* = 0.004; (33, 45); Hospital participants: OR = 4.06, 95% CI: 1.27; 13.05, *p* = 0.019; (29, 30)], indicating that the risk of CI was greater among hospital participants than among community-dwelling participants. The subgroup analyses are presented in Supplementary file 4.

### 3.5 Publication bias assessment

No significant publication bias was observed in the included studies, as indicated by the Begg’s Test (*p* = 0.161). This is portrayed in Supplementary file 1, Table S8. However, observing the funnel plot, a certain publication bias can be observed.

### 3.6 Sensitivity analysis of all studies

**Figure 3:**
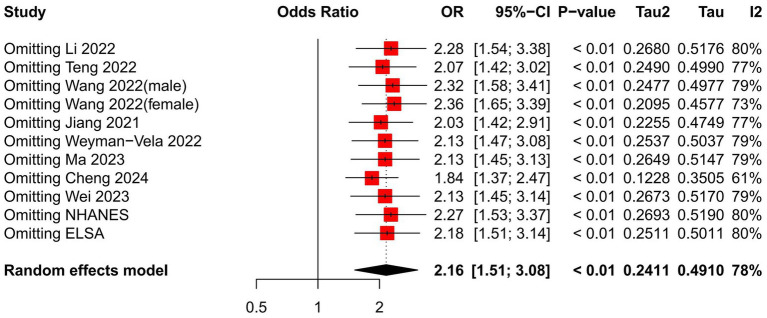
Sensitivity analysis of the pooled results for Group 1.

We performed a sensitivity analysis on the impact of individual studies affecting the relationship between the TyG index and CI by systematically excluding one study at a time and pooling the remaining studies to identify any influential outliers. Figure 3 demonstrates that no statistically significant changes were observed (Table 3).

**Table 3:** Meta-regression for subgroup outcomes of the TyG index in association with cognitive impairment.

Variable	β(95% CI)	SE	*p*
Mean age: ≥65 years vs. <65 years	0.731 (0.086, 1. 375)	0.285	0.030
Study population: community-dwelling participants vs. hospital participants	−1.123(−1.674, −0.571)	0.244	0.001
Study design: Longitudinal study vs. cross-sectional study	0.080(− 0.848, 1.008)	0.410	0.850
Region: Asia vs. other	0.084(− 0.8 6 8, 1.037)	0.421	0.846
Cognitive impairment assessment: MMSE vs. other	0.122(−0.780, 1.024)	0.399	0.766
Type of cognitive disorder: Cognitive Impairment vs. Vascular Cognitive Impairment	−1.163(−1.936,-0.391)	0.341	0.008

SE, Standard Error; TyG index, Triglyceride glucose index; MMSE, Mini Mental State Examination.

## 4 Discussion

Our study is the first to comprehensively search and combine relevant national databases, while focusing on a systematic review and meta-analysis of the association of the TyG index with CI. We found that there was a positive association between TyG index and risk of incident CI which remained stable even after adjustment for covariates. Notably, this correlation appears to be most pronounced in individuals with VCI who have not yet progressed to dementia.

Our study found that the TyG index has potential value in optimizing risk stratification for CI. The TyG index is an efficient and low-cost biomarker used to identify IR, which remains a critical pathophysiology pathway for the development and risk stratification of diabetes, cardiovascular and cerebrovascular disease (27, 47). These three diseases are highly prevalent chronic diseases in the elderly and independently associated with the development of CI as well. It is universally known that diabetes itself is a definitive risk factor for CI, as it increases the risk of neurodegeneration via tau-mediated mechanisms which even precede cerebrovascular injury (20). Secondly, the pathways leading to vascular injury and neurodegeneration may have common underlying mechanisms, such as IR, advanced glycation, and chronic inflammation, which are all related to the pathogenesis of CI (48–50). From the perspective of vascular damage, more studies in recent years have shown a clear correlation between TyG index and the occurrence of cardiovascular and cerebrovascular disease (27, 51, 52). Cerebrovascular diseases related to CI can be divided into two categories: CI after stroke and cerebral small-vessel disease (such as white matter hyperintensities, lacunes, cerebral microbleeds, and enlarged perivascular spaces). These diseases, whether they cause damage to brain structures or functional impairment, are important causes of CI (46, 50, 53). Finally, from the perspective of life care, the elderly with chronic diseases such as diabetes and vascular diseases demonstrate decreased self-care and exercise, and increased social isolation and utilization of nursing services compared to normal elderly, which are all important risk factors and modifiers for CI (20, 47).

The TyG index shows promise as an early screening tool for CI, but its applicability requires further exploration. The type, severity, definitions and measurement methods of CI all directly influence the effectiveness of the TyG index as a screening tool. Our conclusion indicated that the TyG index had a stronger consistency with VCI than non-VCI. As our team previously found, the TyG index has potential value in optimizing risk strategies for ischemic stroke in the general population, and there is a significant association between high TyG index and many adverse outcomes of stroke (27). Ischemic stroke is the most important source of vascular dementia (VD) (54), and the TyG index is likely to affect cerebral vascular reserve by interfering with insulin signaling, enhancing chronic systemic inflammation, increasing the formation of foam cells, and affecting the metabolism of insulin-like growth factors (IGF-1 and IGF-2), cyclic guanosine monophosphate (cGMP), and nitric oxide (NO), thereby further leading to a decline in cognitive.

By further refining subgroup analysis, we found that the consistency between the TyG index and the MoCA scores was higher than that with the MMSE scores. Since MoCA is more sensitive than MMSE in detecting early cognitive decline (55, 56), this suggested that the TyG index may have a higher value in the diagnosis of early cognitive decline. To verify this finding, we conducted subgroup analyses for different cognitive levels and found that the OR for CI was greater than that for definite dementia, further supporting the diagnostic value of the TyG index in early CI, especially non-dementia CI.

Our study also found that hospitalized participants with a high TyG index demonstrated a more significant increase in the risk of CI compared to community participants. This phenomenon may be related to several factors. To begin with, research has proven that any malnutrition or health problem can lead to significant changes in fasting blood glucose levels (57). Impaired fasting glucose may then lead to the accumulation of lipid accumulation products, which in turn can affect fasting blood lipid levels (57, 58). Secondly, the exercise intensity of hospitalized patients is significantly reduced, which can also affect fasting blood glucose and lipid level (57, 59–61). Thirdly, studies have shown that Western diets (WD), represented by high-fat and high cholesterol diets (WD), lead to elevated blood lipids and were a contributing factor to AD. WD may accelerate and enhance inflammation, leading to obesity, metabolic disorders and gut microbiota dysbiosis, further disrupting the blood–brain barrier, causing central nervous system neuroinflammation, amyloid protein, and tau pathology, ultimately leading to a decline in cognitive function in humans (62). Furthermore, hospitalized patients are often suffering from the acute phase of disease processes, which can lead to accelerated cognitive decline compared to chronic, stable disease populations in the community (63, 64).

We observed heterogeneity in effects of TyG index and CI between studies, with estimated values ranging from 1.11 (95% CI = 0.87–1.42) in Wang et al. (41) (women) to 7.37 (95% CI = 4.72–11.50) in Xue Wei Tong et al. (29). This heterogeneity may stem from differences in phenotypic criteria used in the studies, such as inconsistent assessment methods for detecting CI or the TyG index, gender differences, and various other potential factors. Our research indicated that, although the overall outcomes were similar whether the TyG index was considered as a categorical or continuous variable, the TyG index as a categorical variable held greater predictive power in individuals aged over 65, while the continuous variable format was more predictive in those under 65. Notably, in addition to age, as our meta-regression found, the type of population may be the key to heterogeneity. In hospitalized patients, the TyG index associated more positively with CI compared to community populations, which may be attributed to hospitalized patients often suffering from multiple comorbidities, such as hyperlipidemia, cardiovascular disease, cerebrovascular disease, systemic immune inflammation, poor sleep patterns, or depression, all of which may overlap with the original effects (27, 48, 51, 65–67). However, despite this heterogeneity, all results were still found to be consistent, with no studies reporting that the TyG index was a protective factor against the development of CI.

Overall, the CIs of our included studies were found to be fairly robust, though there were two studies which exhibited a greater degree of larger variance in CIs (31, 32). Both were case–control studies, which may have included factors such as recall bias which could have affected the actual data. In addition, there were significant differences in the CIs of the two national databases included in this study, which may be due to differences in the research subjects, cognitive disorder assessment methods, and individual data collection processes of the two databases.

Our study had several advantages: First, the overall sample size was large with inclusion of multiple regions. Next, the association between the TyG index and CI outcomes was not affected by any single inclusion factor or participant selection. All these aspects provide evidence for the stability and credibility of our results presented. Furthermore, we not only included all the latest studies that met the inclusion criteria, but also added data from two relevant authoritative national databases, which improves the reliability of our results even more so. Last but not least, two previous meta-analysis reported an association between TyG index and CI (41, 68), indicating that researchers were gradually emphasizing the diagnostic value of TyG in cognitive function. However, both of these studies have certain flaws. In a study by Wang et al., Hong’s original study included a population of over 5 million people, but in this meta-analysis, a population of over 8 million was included. This huge mistake may lead to a decrease in the credibility of the results. In another study by Yuqing Han, RR (relative risk) was still used instead of OR (odds ratio), and there is no numerical conversion of OR even in cross-sectional study. Therefore, in order to more rigorously complete the correlation analysis between TyG index and CI, we extracted OR as the effect size (69, 70). Although we strictly followed established standards for research selection, data extraction, and quality evaluation, there are still some limitations. To begin with, all included studies were observational studies, which did not allow our analysis to fully demonstrate the causal relationship between TyG index and CI, which would be possible with analysis of randomized clinical trials. Secondly, the study population included mostly Asian subjects, with only two studies from America (44, 45). Although we supplemented with databases from the United States and England, the generalizability of our results remains nonideal. Thirdly, when we employed both Begg’s Regression Test and funnel plot to analyze publication bias, we encountered two contradictory outcomes. This discordance may arise from the inclusion of small-sample studies or due to certain heterogeneities within the studies, as previously mentioned. Future research, particularly large-sample, multicenter studies, is eagerly anticipated to address these issues. Last but not least, the glucose and triglycerides levels included in the TyG index calculation were measured with heterogeneous and non-uniform methods which differed by institution. The cognitive assessment methods used different scales, and there is a limitation in conducting a thorough analysis to accurately determine the impact of genetic predisposition, environmental exposures, and psychosocial factors on the results. These factors may affect the consistency of the data. It is hoped that future original research will delve into these issues to provide a more in-depth exploration.

In conclusion, our systematic review and meta-analysis indicates a correlation between the TyG index and CI. Higher TyG index values are associated with a greater risk of CI in both older adults and those under 65. Importantly, this correlation appears to be most pronounced in individuals with VCI who have not yet progressed to dementia. With the continuous intensification of global aging, early diagnosis of CI is an issue that must be taken seriously. Most neuropsychological assessments of cognition are not sensitive enough or too time-consuming for the diagnosis of early CI and are often influenced by educational level, lifestyle factors, and racial diversity. The TyG index is convenient, affordable, and easily administered, and may play an important role for early risk assessment of CI. Unfortunately, there are relatively few well-designed studies focusing on the TyG index specifically in relation to CI currently. Therefore, more prospective cohort studies should be conducted, including large sample sizes and multicenter clinical trials using the TyG index as a predictor to advance our clinical management and utilization of this biomarker in CI research. In addition, carefully designed basic science studies are required to further explore the potential mechanisms and relationships between the TyG index and CI pathophysiology and associated sequelae.

## Data Availability

The original contributions presented in the study are included in the article/Supplementary material, further inquiries can be directed to the corresponding authors.
