# Supplementary material for: The triglyceride-glucose index and risk of cognitive impairment: a systematic review and meta-analysis with inclusion of two national databases
Source: Front Neurol. 2024 Nov 29;15:1496871. doi: 10.3389/fneur.2024.1496871 (PMC11638587; doi:10.3389/fneur.2024.1496871)
Supplement: Supplementary file 1 [file Table_1.docx]

***Supplementary File One：***

Table S1 Characteristics of the population from the NHANES database.

Table S2 Multivariable logistic regression to assess the association of TYG with Cognitive impairment from NHANES database.

Table S3 Characteristics of the population from the ELSA database.

Table S4 Multivariable logistic regression to assess the association of TYG with Cognitive impairment from ELSA database.

Table S5 The quality assessment of included studies by NOS scale(cohort study).

Table S6 The quality assessment of included studies by NOS scale(case-control study).

Table S7 The quality assessment of included studies by AHRQ scale (cross-sectional study).

Table 8 Publication bias of meta-analysis results.

**Table S1 Characteristics of the population from the NHANES database.**

| **TYG quartile** | **Q1** | **Q2** | **Q3** | **Q4** | **P-value*** |
| --- | --- | --- | --- | --- | --- |
| **N** | 347 | 348 | 348 | 348 |  |
| **AGE（years）** |  |  |  |  | 0.679 |
| **<65** | 113 (32.56%) | 100 (28.74%) | 106 (30.46%) | 101 (29.02%) |  |
| **≥65** | 234 (67.44%) | 248 (71.26%) | 242 (69.54%) | 247 (70.98%) |  |
| **Gender** |  |  |  |  | 0.089 |
| **Male** | 158 (45.53%) | 182 (52.30%) | 160 (45.98%) | 184 (52.87%) |  |
| **Female** | 189 (54.47%) | 166 (47.70%) | 188 (54.02%) | 164 (47.13%) |  |
| **BMI** | 27.26 ± 6.13 | 27.72 ± 5.38 | 29.86 ± 6.54 | 31.53 ± 6.24 | <0.001 |
| **Waist circumference(cm)** | 96.39 ± 14.65 | 98.94 ± 13.37 | 104.11 ± 14.42 | 108.99 ± 13.99 | <0.001 |
| **≥25** | 204 (58.79%) | 235 (67.53%) | 266 (76.44%) | 304 (87.36%) |  |
| **Smoking status** |  |  |  |  | 0.334 |
| **Non-smoker** | 186 (53.60%) | 172 (49.43%) | 173 (49.71%) | 158 (45.40%) |  |
| **Ex-smoker** | 37 (10.66%) | 38 (10.92%) | 49 (14.08%) | 45 (12.93%) |  |
| **Current smoker** | 124 (35.73%) | 138 (39.66%) | 126 (36.21%) | 145 (41.67%) |  |
| **Alcohol** |  |  |  |  | 0.036 |
| **No** | 132 (38.04%) | 153 (43.97%) | 126 (36.21%) | 117 (33.62%) |  |
| **Yes** | 215 (61.96%) | 195 (56.03%) | 222 (63.79%) | 231 (66.38%) |  |
| **SBP (mmHg)** | 125.63 ± 20.70 | 129.38 ± 23.45 | 126.43 ± 22.03 | 131.33 ± 22.08 | 0.003 |
| **DBP (mmHg)** | 64.68 ± 13.62 | 65.36 ± 14.88 | 64.61 ± 13.87 | 66.03 ± 14.32 | 0.440 |
| **Glucose (mg/dl)** | 99.13 ± 13.21 | 105.62 ± 16.15 | 114.29 ± 27.20 | 142.66 ± 52.50 | <0.001 |
| **Total cholesterol (mg/dl)** | 181.13 ± 37.59 | 190.45 ± 42.04 | 191.39 ± 42.40 | 198.59 ± 44.77 | <0.001 |
| **HDL (mg/dl)** | 67.15 ± 19.21 | 57.97 ± 15.31 | 51.71 ± 12.16 | 45.67 ± 10.82 | <0.001 |
| **LDL (mg/dl)** | 101.84 ± 29.95 | 114.18 ± 35.63 | 114.13 ± 37.58 | 111.94 ± 39.27 | <0.001 |
| **Triglyceride (mg/dl)** | 60.67 ± 15.27 | 91.66 ± 14.76 | 127.70 ± 24.57 | 209.72 ± 83.27 | <0.001 |
| **Diabetes** |  |  |  |  | <0.001 |
| **No** | 305 (87.90%) | 277 (79.60%) | 233 (66.95%) | 152 (43.68%) |  |
| **Yes** | 42 (12.10%) | 71 (20.40%) | 115 (33.05%) | 196 (56.32%) |  |
| **Hypertension** |  |  |  |  | <0.001 |
| **No** | 145 (41.79%) | 130 (37.36%) | 114 (32.76%) | 85 (24.43%) |  |
| **Yes** | 202 (58.21%) | 218 (62.64%) | 234 (67.24%) | 263 (75.57%) |  |
| **Dyslipidemia** |  |  |  |  | <0.001 |
| **No** | 144 (41.50%) | 97 (27.87%) | 91 (26.15%) | 67 (19.25%) |  |
| **Yes** | 203 (58.50%) | 251 (72.13%) | 257 (73.85%) | 281 (80.75%) |  |
| **CKD** |  |  |  |  | 0.071 |
| **No** | 140 (40.35%) | 142 (40.80%) | 129 (37.07%) | 112 (32.18%) |  |
| **Yes** | 207 (59.65%) | 206 (59.20%) | 219 (62.93%) | 236 (67.82%) |  |
| **IR** | 25.77 ± 6.63 | 24.48 ± 6.60 | 24.41 ± 6.34 | 24.96 ± 6.42 | 0.012 |
| **DSST** | 47.34 ± 17.79 | 45.11 ± 16.92 | 44.39 ± 17.38 | 44.47 ± 17.64 | 0.108 |
| **AFT** | 16.87 ± 5.62 | 16.52 ± 5.43 | 16.61 ± 5.51 | 16.44 ± 5.21 | 0.689 |

Abbreviations: BMI, Body Mass Index; SBP, Systolic blood pressure; DBP, diastolic blood pressure; HDL, high-density lipoprotein; LDL, low-density lipoprotein; CKD, chronic kidney disease; IR, immediate verbal list learning; DSST, digital symbol substitution test; AFT, animal fluency test.

**Table S2 Multivariable logistic regression to assess the association of TyG index** **with Cognitive impairment from NHANES database.**

|  | **Model 1** | | **Model 2** | | **Model 3** | |
| --- | --- | --- | --- | --- | --- | --- |
| **TYG quartil**e | OR (95% CI) | P value | OR (95% CI) | P value | OR (95% CI) | P value |
| **Q1** | 1.0 |  | 1.0 |  | 1.0 |  |
| **Q2** | 0.84 (0.59, 1.20) | 0.3332 | 0.89 (0.62, 1.27) | 0.5204 | 1.17 (0.81, 1.68) | 0.4144 |
| **Q3** | 0.85 (0.60, 1.22) | 0.3789 | 0.84 (0.58, 1.20) | 0.3298 | 1.24 (0.86, 1.78) | 0.2570 |
| **Q4** | 0.72 (0.51, 1.02) | 0.0681 | 0.72 (0.51, 1.03) | 0.0710 | 1.53 (1.06, 2.20) | 0.0235 |

Model1 was no adjusted.

Model2 was adjusted for age, gender.

Model3 was adjusted for age, gender, CKD, smoking, Alcohol, Dyslipidemia.

**Table S3 Characteristics of the population from the ELSA database.**

| **TYG group** | **Q1** | **Q2** | **Q3** | **Q4** | **P value** |
| --- | --- | --- | --- | --- | --- |
| **N** | 396 | 406 | 403 | 402 |  |
| **Age** | 62.23 ± 6.48 | 63.46 ± 6.39 | 63.25 ± 6.09 | 62.57 ± 6.41 | 0.012 |
| **Gender** |  |  |  |  | 0.008 |
| **Male** | 150 (37.88%) | 164 (40.39%) | 171 (42.43%) | 198 (49.25%) |  |
| **Female** | 246 (62.12%) | 242 (59.61%) | 232 (57.57%) | 204 (50.75%) |  |
| **Total cholesterol** | 5.36 ± 1.05 | 5.61 ± 1.13 | 5.71 ± 1.15 | 5.83 ± 1.22 | <0.001 |
| **HDL** | 2.02 ± 0.50 | 1.82 ± 0.44 | 1.61 ± 0.36 | 1.38 ± 0.33 | <0.001 |
| **LDL** | 3.00 ± 0.88 | 3.31 ± 1.00 | 3.47 ± 1.04 | 3.45 ± 1.10 | <0.001 |
| **BMI** | 25.84 ± 4.35 | 27.18 ± 4.61 | 28.73 ± 4.91 | 29.77 ± 4.75 | <0.001 |
| **Alcohol** |  |  |  |  | 0.280 |
| **No** | 22 (5.56%) | 12 (2.96%) | 21 (5.21%) | 17 (4.23%) |  |
| **Yes** | 374 (94.44%) | 394 (97.04%) | 382 (94.79%) | 385 (95.77%) |  |
| **Smoke** |  |  |  |  | 0.072 |
| **No** | 370 (93.43%) | 369 (90.89%) | 373 (92.56%) | 356 (88.56%) |  |
| **Yes** | 26 (6.57%) | 37 (9.11%) | 30 (7.44%) | 46 (11.44%) |  |
| **Physical activity** |  |  |  |  | 0.007 |
| **No** | 38 (9.60%) | 61 (15.02%) | 67 (16.63%) | 71 (17.66%) |  |
| **Yes** | 358 (90.40%) | 345 (84.98%) | 336 (83.37%) | 331 (82.34%) |  |
| **Hypertension** |  |  |  |  | <0.001 |
| **No** | 302 (76.26%) | 299 (73.65%) | 276 (68.49%) | 256 (63.68%) |  |
| **Yes** | 94 (23.74%) | 107 (26.35%) | 127 (31.51%) | 146 (36.32%) |  |
| **Diabetes** |  |  |  |  | <0.001 |
| **No** | 391 (98.74%) | 399 (98.28%) | 395 (98.01%) | 376 (93.53%) |  |
| **Yes**  **Cognitive impairment** | 5 (1.26%) | 7 (1.72%) | 8 (1.99%) | 26 (6.47%) | 0.558 |
|  |  |  |  |  |  |
| **No** | 394 (99.49%) | 402 (99.01%) | 402 (99.75%) | 400  (99.50%) |  |
| **Yes** | 2 (0.51%) | 4 (0.99%) | 1 (0.25%) | 2 (0.50%) |  |

Abbreviations: HDL, high-density lipoprotein; LDL, low-density lipoprotein; BMI, Body Mass Index.

**Table S4 Multivariable logistic regression to assess the association of TyG index with Cognitive impairment from ELSA database.**

|  | **Model 1** | | **Model 2** | | **Model 3** | |
| --- | --- | --- | --- | --- | --- | --- |
| **TYG quartil**e | OR (95% CI) | P value | OR (95% CI) | P value | OR (95% CI) | P value |
| **Q1** | 1.0 |  | 1.0 |  | 1.0 |  |
| **Q2** | 1.96 (0.36, 10.76) | 0.4386 | 1.81 (0.33, 9.97) | 0.4963 | 2.46 (0.43, 14.03) | 0.3097 |
| **Q3** | 0.49 (0.04, 5.43) | 0.5609 | 0.46 (0.04, 5.13) | 0.5297 | 0.66 (0.06, 7.55) | 0.7381 |
| **Q4** | 0.99 (0.14, 7.03) | 0.9880 | 0.96 (0.13, 6.89) | 0.9707 | 1.56 (0.21, 11.67) | 0.6667 |

**Table S5 The quality assessment of included studies by NOS scale(cohort study).**

|  |  | Selection | | | | Comparability | Outcome | | | Score |
| --- | --- | --- | --- | --- | --- | --- | --- | --- | --- | --- |
| NO. | Study | Representativeness of the intervention cohort. | Selection of the non intervention cohort. | Ascertainment of intervention. | Demonstration that outcome of interest was not present at start of study. | Whether significant confounders were adjusted for. | Assessment of outcome. | Was follow up long enough for outcomes to occur. | Adequacy of follow up of cohorts. | Total  points |
| 1 | Hong 2021 | ★ | ★ | ★ | ★ | ★★ | ★ | ★ | ★ | 9 |
| 2 | Li 2022 | ★ | ★ | ★ | ★ | ★★ | ★ | ★ | ★ | 9 |
| 3 | Teng 2022 |  | ★ | ★ | ★ | ★★ | ★ | ★ | ★ | 8 |
| 4 | Wang 2022 | ★ | ★ | ★ |  | ★★ | ★ | ★ | ★ | 8 |
| 5 | Faqih 2021 |  | ★ | ★ |  | ★★ | ★ | ★ | ★ | 7 |
| 6 | Cheng 2024 | ★ | ★ | ★ | ★ | ★★ | ★ | ★ | ★ | 9 |
| 7 | Sun 2023 | ★ | ★ | ★ | ★ | ★★ | ★ | ★ | ★ | 9 |

**Table S6 The quality assessment of included studies by NOS scale(case-control study).**

|  |  | Selection | | | | Comparability | Outcome | | | Score |
| --- | --- | --- | --- | --- | --- | --- | --- | --- | --- | --- |
| NO. | Study | Is the definition of a case adequate? | Are the cases representative? | Selection of controls. | Definition of Contrast. | Comparability of cases and controls based on the design or analysis obtained. | Determinati-on of exposure. | Whether exposures of cases and controls were determined using the same method. | Non-response rate. | Total  points |
| 1 | Jiang 2021 | ★ |  | ★ |  | ★★ | ★ | ★ |  | 6 |
| 2 | Weyman-Vela 2022 | ★ | ★ |  | ★ | ★★ | ★ | ★ | ★ | 8 |

**Table S7** **The quality assessment of included studies by AHRQ scale (cross-sectional study).**

| NO. | Study | Criteria  1 | Criteria  2 | Criteria  3 | Criteria  4 | Criteria  5 | Criteria  6 | Criteria  7 | Criteria  8 | Criteria  9 | Criteria  10 | Criteria  11 | Quality |
| --- | --- | --- | --- | --- | --- | --- | --- | --- | --- | --- | --- | --- | --- |
| 1 | Tong 2022 | Yes | Yes | Yes | No | No | Yes | Yes | Yes | No | Unclear | No | Medium |
| 2 | Ma 2023 | Yes | Yes | Yes | Yes | No | Yes | Yes | Yes | Yes | Unclear | No | High |
| 3 | Tian 2023 | Yes | Yes | Yes | Yes | No | Yes | Yes | Yes | No | Unclear | No | Medium |
| 4 | Wei 2023 | Yes | Yes | Yes | Yes | No | No | Yes | Yes | No | Unclear | No | Medium |

Quality of the selected observational study was assessed using the Agency for Healthcare Research and Quality(AHRQ) for cross-sectional studies. Criteria.1.Define the source of information (survey, record review)? Criteria.2.List inclusion and exclusion criteria for exposed and unexposed subjects (cases and controls) or refer to previous publications? Criteria3.lndicate time period used for identifying patients? Criteria4.Indicate whether or not subjects were consecutive if not population-based? Criteria5. Indicate if evaluators of subjective components of study were masked to other aspects of the status of the participants? Criteria6. Describe any assessments undertaken for quality assurance purposes (e.g. testretest of primary outcome measurements)? Criteria7. Explain any patient exclusions from analysis? Criteria8. Describe how confounding was assessed and/or controlled? Criteria9. lf applicable, explain how missing data were handled in the analysis? Criteria10. Summarize patient response rates and completeness of data collection? Criteria11. Clarify what follow-up, if any, was expected and the percentage of patients for which incomplete dataor follow-up was obtained?" Yes" was given a point, "No" or "unclear" was not given a point. Studies with a total score of 8-11 were considered high quality, 4-7 were medium quality, and 0-3 were low quality.

**Table S8 Publication bias of meta-analysis results.**

| **Study** | **z** | **Begg's test** |
| --- | --- | --- |
| **All studies** | 1.40 | 0.161 |
