# Supplementary material for: The triglyceride-glucose index and risk of cognitive impairment: a systematic review and meta-analysis with inclusion of two national databases
Source: Front Neurol. 2024 Nov 29;15:1496871. doi: 10.3389/fneur.2024.1496871 (PMC11638587; doi:10.3389/fneur.2024.1496871)
Supplement: Supplementary file 2 [file Table_2.docx]

**Supplementary File 2. The results of subgroup analysis of the correlation between TyG index and cognitive impairment of Group2**

| **Variable** | **N** | **Meta-analysis results** | **P(z-text)** | **Heterogeneity** |
| --- | --- | --- | --- | --- |
| **Total** | 4 | 2.58 (1.27, 5.22) | 0.009 | I^2^=90.0%, P<0.01 |
| **Mean age** |  |  |  |  |
| ≥65 years | 2 | 1.92 (1.39, 2.65) | <0.001 | I^2^=1.4%, P=0.314 |
| <65 years | 2 | 3.48 (0.80, 15.19) | 0.097 | I^2^=95.1%, P=0.000 |
| **Study population** |  |  |  |  |
| Community-dwelling participants | 2 | 1.62 (1.17, 2.27) | 0.004 | I^2^=0.0%, P=0.957 |
| Hospital participants | 2 | 4.06 (1.27, 13.05) | 0.019 | I^2^=92.8%, P=0.000 |

Abbreviations: OR, Odds Raito; TyG index, Triglyceride glucose index.
