# Supplementary material for: The triglyceride-glucose index and risk of cognitive impairment: a systematic review and meta-analysis with inclusion of two national databases
Source: Front Neurol. 2024 Nov 29;15:1496871. doi: 10.3389/fneur.2024.1496871 (PMC11638587; doi:10.3389/fneur.2024.1496871)
Supplement: Supplementary file 3 [file Table_3.docx]

**Supplementary File 3. Meta-regression for subgroup outcomes of the TyG index in association with cognitive impairment.**

| **Variable** | **β(95% CI)** | **SE** | **p** |
| --- | --- | --- | --- |
| Mean age: ≥65 years vs <65 years | -0.608(-3.917, 2.701) | 0.796 | 0.512 |
| Study population: community-dwelling participants vs hospital participants | -0.916(-3.500, 1.667) | 0.600 | 0.267 |

Abbreviations: SE, Standard Error; TyG index, Triglyceride glucose index;
